# Low Frequency Systemic Hemodynamic “Noise” in Resting State BOLD fMRI: Characteristics, Causes, Implications, Mitigation Strategies, and Applications

**DOI:** 10.3389/fnins.2019.00787

**Published:** 2019-08-14

**Authors:** Yunjie Tong, Lia M. Hocke, Blaise B. Frederick

**Affiliations:** ^1^Weldon School of Biomedical Engineering, Purdue University, West Lafayette, IN, United States; ^2^McLean Imaging Center, McLean Hospital, Belmont, MA, United States; ^3^Department of Psychiatry, Harvard Medical School, Boston, MA, United States

**Keywords:** low frequency oscillation, noise modeling, denoising, vascular mapping, cerebrovascular reactivity, physiological noise, physiological noise modeling

## Abstract

Advances in functional magnetic resonance imaging (fMRI) acquisition have improved signal to noise to the point where the physiology of the subject is the dominant noise source in resting state fMRI data (rsfMRI). Among these systemic, non-neuronal physiological signals, respiration and to some degree cardiac fluctuations can be removed through modeling, or in the case of newer, faster acquisitions such as simultaneous multislice acquisition, simple spectral filtering. However, significant low frequency physiological oscillation (∼0.01–0.15 Hz) remains in the signal. This is problematic, as it is the precise frequency band occupied by the neuronally modulated hemodynamic responses used to study brain connectivity, precluding its removal by spectral filtering. The source of this signal, and its method of production and propagation in the body, have not been conclusively determined. Here, we summarize the defining characteristics of the systemic low frequency noise signal, and review some current theories about the signal source and the evidence supporting them. The strength and distribution of the systemic LFO signal make characterizing and removing it essential for accurate quantification, especially for resting state connectivity, when no stimulation can be compared with the signal. Widespread correlated non-neuronal signals obscure and distort the more localized patterns of neuronal correlations between interacting brain regions; they may even cause apparent connectivity between regions with no neuronal interaction. Here, we discuss a simple method we have developed to parse the global, moving, blood-borne signal from the stationary, neuronal connectivity signals, substantially reducing the negative correlations that result from global signal regression. Finally, we will discuss some of the uses to which the moving systemic low frequency oscillation can be put if we consider it a “signal” carrying information, rather than simply “noise” complicating the interpretation of resting state connectivity. Properly utilizing this signal may offer insights into subtle hemodynamic alterations that can be used as early indicators of circulatory dysfunction in a number of neuropsychiatric conditions, such as prodromal stroke, moyamoya, and Alzheimer’s disease.

## Introduction

Resting state functional magnetic resonance imaging (rsfMRI) seeks to elucidate neuronal connectivity throughout the brain by examining of fMRI signal correlations between regions during scans when the brain is not performing any specific task (it is “at rest”). However, the BOLD signal does not measure neuronal activation directly. It is a composite measurement of hemodynamic properties of blood flow, volume, and oxygenation changes in response to neuronal activity (Buxton et al., 1998) (i.e., neurovascular coupling). In short, increased neuronal firing leads to an increase in regional blood flow, which brings an oversupply of oxygenated blood (Fox and Raichle, 1986). The increase in blood flow and oxygenation leads to an elevation of the BOLD signal. As a result, the observable BOLD signal (blood-related) is much slower (∼s) than neuronal firing (∼ms) (Logothetis et al., 2001) and cannot reflect fast changes of the field potential of neuronal firings. In mathematical terms, the BOLD signal is the result of the convolution of the fast neuronal signals with the slow hemodynamic functions. Therefore, the frequencies of “neuronal” BOLD signals are generally below 0.15 Hz (Josephs and Henson, 1999).

**FIGURE 1:**
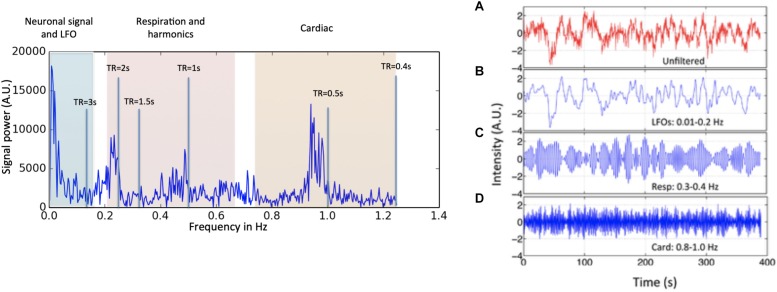
Power spectrum (left) and time domain data (right) presented in different spectral bands, from a voxel in a resting state data (TR = 0.4 s) of one participant. Three distinct spectral ranges corresponding to different physiological processes were marked. The spectral area captured by various TR values is also depicted on the power spectrum. The right hand panel shows a BOLD timecourse from a resting state fMRI scan (TR = 0.4 s) without filtration (**A**, in red) and its band-passed versions in panel **(B)** 0.01–0.2 Hz; **(C)** 0.3–0.4 Hz; and **(D)** 0.8–1.0 Hz (in blue) (Figure adapted from Tong and Frederick, 2014a).

However, neuronal activations are not the only contributors to BOLD signals in the low frequency band. Advances in fMRI acquisition techniques and hardware have improved signal to noise to the point where the physiology of the subject being studied is the dominant noise source in rsfMRI data. In addition to neuronal BOLD, there are systemic, non-neuronal fluctuations in brain hemodynamics due to heartbeat, respiration, and so-called “low frequency oscillations” (LFOs). These signals are unavoidable, and taken together can account for 20–70% of the BOLD signal variance (see Figure 1), depending on acquisition, and locations of the voxels (Liu, 2017). Numerous processing strategies have been devised to mitigate them. Recent improvements in hardware and pulse sequences (specifically simultaneous multislice acquisition protocols) have pushed the temporal resolution of fMRI high enough that respiration, and to some degree cardiac fluctuations (see Figure 1) can be removed through simple spectral filtering, or through more advanced modeling methods when these signals are aliased (Glover et al., 2000; Birn et al., 2006, 2008; Behzadi et al., 2007; Chang et al., 2009, 2013). These methods are well described elsewhere and are not the focus of this manuscript. However, significant signal power remains in the “low frequency oscillation band,” a loosely defined region from ∼0.01–0.15 Hz. Non-neuronal signal in this frequency band accounts for at least 30% of the signal variance in gray matter (Frederick et al., 2012a). This is problematic as it is the precise frequency band occupied by the neuronally modulated hemodynamic responses used to study brain connectivity, precluding its removal by filtering.

The strength and distribution of the systemic LFO signal make characterizing and removing it essential for accurate quantification of neuronal connectivity. Moreover, these signals might not be “noise” after all. Understanding their origins and characteristics will help to develop novel methods to assess brain physiology which could greatly compliment the functional findings.

## Characteristics of Low Frequency Oscillations

LFOs in BOLD fMRI have been found and studied extensively, but as noted above, there are numerous potential explanations for this signal, and even some variation in what in particular should be considered a “low frequency oscillation.” For the remainder of this paper, we will apply two criteria to our discussions of LFOs; the frequency band of the signal, and whether the signal is stationary, or moves with the blood.

### Frequency Content

The first criterion is simply a definition. LFOs are signals that occur in the brain (and in some cases throughout the body) that have frequencies between ∼0.009 and 0.2 Hz. The exact endpoints of this band are extremely variable in the literature. Biswal’s original paper on resting state connectivity used 0.01–0.1 Hz (Biswal et al., 1995), but later papers expanded this range; for the purpose of this discussion, we will use the range of 0.01–0.15. 0.15 Hz has a particular significance as the top of the range, as this is the highest expected frequency in neuronally generated hemodynamic signals (based on the shape of the canonical hemodynamic response function) (Josephs and Henson, 1999), so this defines the frequency range where spectral filtering cannot be used to remove non-neuronal signal. The frequency content of a typical BOLD signal is shown in Figure 1.

### Dynamic Versus Stationary Noise Signals

The second defining characteristic of LFOs is less commonly considered. Our research into low frequency physiological noise in fMRI has established that a significant fraction of the low frequency variance in fMRI data can be modeled quite effectively as a single low frequency signal with varying delay times across the brain. Moreover, the pattern of relative delay times in different regions of the brain is consistent with the delays that would be expected if the signal were moving through the brain with blood as it flowed through the vasculature. We refer to this dynamic signal as “systemic low frequency oscillations” (sLFOs) (Tong et al., 2015). The realization that a significant fraction of the low frequency “noise” in fMRI appears to be moving has important implications for how to identify, remove, or even utilize this signal (Tong and Frederick, 2010, 2012, 2014b; Tong et al., 2011b,c, 2013, 2014, 2017, 2018; Frederick et al., 2012b).

### Temporal Pattern – sLFOs Propagate on a Hemodynamic Timescale

Our research on sLFOs strongly suggest that the underlying oscillations propagate on hemodynamic, physiological, rather than on neuronal, timescales, taking several seconds to fully transit the brain rather than milliseconds. Circulatory measurements of the traversal of a Tc99 tracer through the brain vasculature (from the carotid, to the internal brain arteries, through the parenchyma, to the superior sagittal sinus) showed that the transit time of blood from the anterior and middle cerebral arteries to the superior sagittal sinus takes ∼6.7 s in healthy middle-aged controls (Crandell et al., 1973), timing consistent with results found by other imaging methods. For example, using echo contrast-enhanced ultrasound, delays of 7.5 ± 1.8 s from the carotids to the jugulars were found in 64 healthy subjects (Schreiber et al., 2002). Similar, but smaller delays (4.9–6.4 s) were found in other ultrasound studies (Schreiber et al., 2005). Direct evidence can also be found in digital subtraction angiography (DSA), where an x-ray contrast bolus was injected directly into the ICA and followed as it passed through the brain (Monti et al., 2015; Jann et al., 2016).

### Spatial Pattern – sLFOs Travel Along the Vasculature

The patterns of the delays of the sLFO signal clearly suggest that this signal is related to blood flow, evolving in a pattern that reflects the vasculature – the sLFO signal appears first in the center of the brain, propagates out through the parenchyma, and ends up in the superior sagittal sinus, with a range of delays of ∼6.5 s (Tong and Frederick, 2010), a pattern we have seen consistently in many subsequent studies over several years (Tong et al., 2011a,b,c, 2013, 2014, 2015; Frederick et al., 2012a; Tong and Frederick, 2012, 2014a). In our most recent studies we have directly confirmed the association between the sLFO delay pattern and blood flow, first by performing sLFO analysis and time resolved dynamic susceptibility imaging in the same scan session (Tong et al., 2017), and more recently by following the sLFO signal all the way from the internal carotid arteries through the draining veins (Tong et al., 2018).

### Origin – sLFOs Seem to Originate Outside the Brain

The sLFO BOLD signal identified from the carotids preceded the signal found in any voxel of the brain. Indeed, the same sLFO signal can be found throughout the body (measured in the periphery using NIRS) (Frederick and Tong, 2010; Tong and Frederick, 2010; Li et al., 2018). The delays in the periphery are symmetric across the midline of the body, and the arrival time of the sLFO signal found in the fingers and toes precede the arrival in many brain voxels (Tong et al., 2012). While it is possible that some process in the brain is the ultimate source of this moving signal, there is no evidence whatsoever in the fMRI data that this is the case – to the contrary the implication is that the signal does *not* originate in the brain.

### Summary

From the growing body of evidence from our group and others we can summarize that the sLFO BOLD signal: (1) is a spontaneous physiological oscillation, (2) travels with the blood, and (3) has an extracerebral origin. Given the large amount of LFO signal variance that is clearly attributable to the moving component (at least 30% of the low frequency signal variance in gray matter, Frederick et al., 2012a), we believe this constitutes the majority of the physiological LFO signal power. In addition, these qualities provide the key to isolating the signal from the neuronal signals of interest.

In contrast, there may also be non-neuronal LFOs which do not propagate. However, because of the difficulty in separating these signals from putative neuronal signals, they are far harder to characterize. Certain mechanisms (detailed below) such as the Mayer wave, are thought to be synchronous throughout the body, and therefore stationary. Isolating the contribution of stationary LFOs to the resting state signal would require as yet undeveloped processing strategies, which is why we will discuss these possible sources, however we will focus primarily on the dynamic portion of the signal during the remainder of the paper.

## Potential Causes of the Low Frequency Oscillation

The source of LFO signal, and its mechanism of production and propagation in the body, have not been conclusively determined – LFOs have been variously attributed to alterations in sympathetic nervous system tone, partial pressure of carbon dioxide (paCO_2_) fluctuations modulated by respiration, blood pressure regulation, low frequency neuronal “waves,” and even gastric motility. It may in fact be a combination of multiple, independent signals with distinct sources. We will review these current theories about the signal source and the evidence supporting them. This section is summarized in Table 1.

**TABLE 1:** Summary of the common explanations for low frequency physiological noise.

**Source**	**Description**	**Properties**	**Cerebral origin?**	**Causes sLFO?**
Mayer wave	Mayer waves are spontaneous LFOs at around 0.1 Hz.	Highly synchronous within the brain – it seems unlikely that it would appear to move.	Yes?	Not likely
Vasomotion	Vasomotion is a spontaneous oscillation (0.01∼0.3 Hz) in the vascular tone, which is independent of respiration, pulsation and neuronal activity.	The induced vascular variations could propagate with the blood, resulting in a mix of stationary and moving signal.	Unclear	Possible
CO_2_	Carbon dioxide is a potent vassal dilator. It can travel with the blood and induce changes in cerebral blood flow and volume.	Clearly a moving signal – the CO_2_ travels in the blood.	No	Possible
Variations in heart rate and respiratory volumes	The variations of the heart rate and respiration, including the depth of the respiration, are in the low frequency range.	Depth of respiration and heart rate variation can alter blood volume (through CO_2_ and pressure changes), and the effects should move with the blood.	No	Possible
Gastric oscillations	The electrogastrogram signal (i.e., synchronized gut motions at ∼0.05 Hz) significant correlates with BOLD fMRI data with time delays.	May be controlled neuronally, but the effects seem to originate in the gut and move with blood.	Mostly no	Possible
Aliased signals of cardiac and respiration	Aliased signals of cardiac and respiration due to long TR are in the low frequency range.	These signals are in the right frequency range and can travel with blood, but are not highly correlated with the sLFO signal when tested by fMRI data with very short TR or fNIRS.	No	Not likely

### Variations in Heart Rate and Respiratory Volumes

One major potential source of LFOs comes from variations of the heart rate and respiration. For example respiration volume per time (RVT) (Birn et al., 2006) and respiration variation (convolved with the respiratory response function, Birn et al., 2008; Chang et al., 2009) are two methods to model fluctuations from CO_2_ concentration. In specific, the former model describes the depth of the respiration, whereas the latter reflects the variation in respiration. Previously, these methods have been shown to explain additive variation in data with longer TRs between 9 and 11% (Birn et al., 2006, 2008; Chang et al., 2009) in the voxels affected. From the same group a model taking the variation in heart rate into account was developed (Chang et al., 2009), explaining 3% more variance in the affected voxels. The underlying mechanism of this change is still not well understood, but was proposed to relate to neuronal activity linked with changes in levels of arousal (Chang et al., 2009). These models typically incorporate a delay of several seconds to best match the modeled noise waveforms with the fMRI data, suggesting that these signals are in the moving category (although the standard implementations of these methods do not account for regional delays within the brain).

### Carbon Dioxide

Carbon dioxide changes are closely related to the previous topic, and they partially share the same mechanism, namely induced changes in cerebral blood flow and volume due to CO_2_ induced vasodilation. However, here we discuss the direct effects of paCO_2_ by comparing BOLD fMRI to measured fluctuations in the partial pressure of end-tidal carbon dioxide, especially in the lower range of the LF band, namely 0–0.05 Hz (Wise et al., 2004; Sassaroli et al., 2012), rather than the indirect estimation of this effect from the respiratory and/or cardiac waveforms.

Wise et al. (2004) found that paCO_2_ levels measured with end tidal CO_2_ in the 0–0.05 Hz band were significantly correlated with both increased middle cerebral artery blood velocity and increased BOLD fMRI signal in gray and white matter. This is attributed to the vasodilatory effect of CO_2_ – increased CO_2_ leads to increased arterial diameter and blood volume. As a consequence, this signal can be clearly placed in the group of sLFOs that move with the blood. Wise found up to 28% of the low frequency signal variance in the BOLD signal was attributable to the paCO_2_. This is likely the lower limit, because while the peak correlation delays between the BOLD and paCO_2_ timecourses were between 3 and 13 s, only a single delay of 6.3 s was used for all voxels, which would reduce the apparent correlation.

### Mayer Waves

Mayer waves are spontaneous LFOs at around 0.1 Hz (Julien, 2006) and are associated with variations in mean arterial pressure, and have been associated with a sympathetic autoregulation mechanism (Tsuji et al., 2000), in particular below 0.1 Hz (Obrig et al., 2000; van Beek et al., 2008; Sassaroli et al., 2012). These studies connected mean arterial blood pressure to cerebral intravascular oxygenation when monitoring cerebral circulation and blood flow with NIRS. However, a characteristic of Mayer waves is their largely synchronous nature within the brain (Sassaroli et al., 2012), differing from the sLFOs, which have differing delays across the brain (Tong and Frederick, 2010; Tong et al., 2011b). We would therefore classify Mayer waves as stationary LFO signals.

### Vasomotion From Oscillations in the Vascular Tone

Vasomotion is a spontaneous oscillation (0.01∼0.3 Hz) in the vascular tone, which is independent of respiration, pulsation and neuronal activity (Hundley et al., 1988; Mayhew et al., 1996; Rivadulla et al., 2011). These oscillations of the lumen diameter modify blood flow in a corresponding fashion resulting in periodic oscillations in the blood flow (Aalkjær et al., 2011). Another LF signal is attributed to vasomotion, referring to the oscillations in the vascular tone, thought to be generated movement within the vessel walls (Zhang et al., 1998; Aalkjær et al., 2011; Sassaroli et al., 2012; Müller and Österreich, 2014). These changes are highly localized and have been linked to oscillatory intracellular calcium (Aalkjær et al., 2011). This would give the signals a local, stationary nature, however the vascular variations induced would then propagate with the blood, resulting in a mix of stationary and moving signal.

### Aliased Signals of Cardiac and Respiration

Because fMRI is usually not sampled fast enough to resolve cardiac or respiratory waveforms, some fraction of the energy in these signals will be aliased to the low frequency band. In order to determine the significance of this component of the signal, we evaluated sLFOs in a dataset with high temporal resolution in which the respiration and cardiac waveforms are fully sampled (Hocke et al., 2016). We found that even with fully sampled data, in which the respiration and cardiac bands can be isolated with spectral filters, the purely non-neuronal sLFOs (as determined by time-delayed correlation with a peripheral NIRS signal) still account for over 13% of the total BOLD signal variance across all frequency bands.

### Gastric Oscillations

A somewhat more recent theory for the cause of sLFOs is proposed by Rebollo et al. (2018) who found significant correlations between electrogastrogram signals (which measure synchronized gut motions at ∼0.05 Hz) and BOLD fMRI data. Unfortunately, the technique used was unable to determine directionality. However, the authors found delays between the earliest (somatosensory cortices) and the latest (dorsal precuneous and extrastriate body area) nodes of the proposed “gastric network” were about 3.3 s. These later regions lie in close proximity to the superior sagittal and transverse sinuses, respectively, vessels at the end of the vascular path through the brain. While Rebollo’s analysis could not ascertain directionality – this observation is consistent with a hemodynamic perturbation generated in the stomach which then propagated through the cerebral vasculature, which suggests that gastric signals likely contribute to the “moving signal” category.

This finding is in good agreement with previous work by Yacin et al. (2011) which showed a strong relationship between gastric activity and systemic LFOs in the periphery. Yacin was able to reconstruct the gastric slow wave signal from a fingertip photoplethysmogram, using a deep learning approach. The reconstructed signal correlated with the measured electrogastrogram slow wave with *R* >= 0.9, clearly establishing that the gastric signal contributed a significant portion of the sLFO variance observable in the periphery.

## Implications for Resting State Analysis

As mentioned previously, BOLD fMRI infers neuronal activation indirectly through neurovascular coupling. As a result, the neuronal activation will appear in the low frequency range (∼0.01–0.15 Hz) of BOLD signal in both resting state and task fMRI studies. This frequency range significantly overlaps with that of sLFO. Therefore, the presence of sLFO in the BOLD signal will confound the results of fMRI analyses, especially in resting state studies, as the neuronal firing is also spontaneous and of unknown timecourse (like sLFO), unlike task activation which can be modeled.

### Pure Physiological sLFOs in Resting State Networks

In the following, we describe a previously published study demonstrating the confounding effect of sLFO to the resting state network analysis in both simulation as well as using concurrent fMRI/fNIRS data (Tong et al., 2013). The signals measured by NIRS (concentration changes in oxyhemoglobin and deoxy-hemoglobin: Δ[HbO] and Δ[Hb]) are, like BOLD fMRI signal, blood-related measures. High consistency between these NIRS and fMRI signals has been demonstrated in concurrent studies (Strangman et al., 2002; Sassaroli et al., 2006; Cui et al., 2011). In this particular concurrent study, Δ[HbO] and Δ[Hb] were measured in the periphery (i.e., on finger and toes) using NIRS, instead of the brain. We found that the LFO band component of Δ[HbO] in the periphery was highly correlated with the sLFOs of the BOLD signal in the brain, with a time delay. Most importantly, since the data was recorded in the periphery, the sLFOs measured here represented “pure” physiological fluctuations that were clearly not contaminated by the neuronal LFO as they would be in the brain.

**FIGURE 2:**
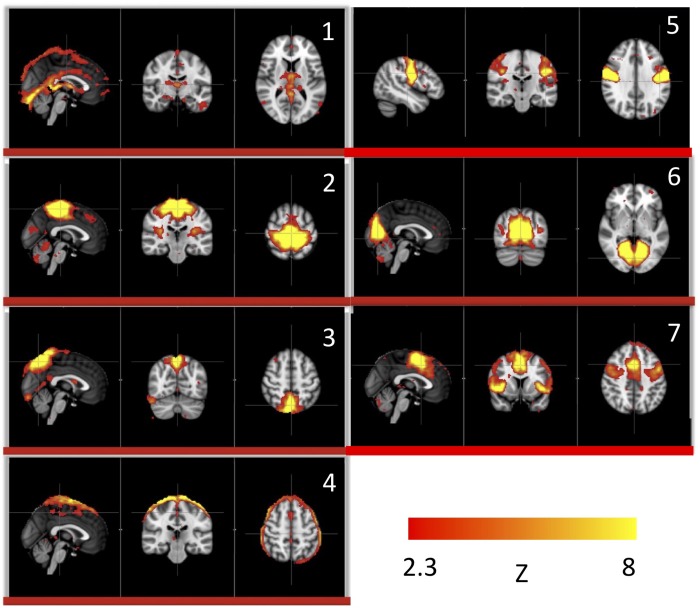
Independent components (1–7) from a group analysis of 10 subjects’ resting state data that have high, significant positive correlations with simultaneously recorded peripheral NIRS data (Figure adapted from Tong et al., 2015).

We used this pure signal to assess the physiological influence of sLFOs on each resting state network. In detail, each subject’s connectivity networks were identified from rsfMRI data using ICA (Melodic and FSL), from which, the signature BOLD signals of each network were extracted. To assess the sLFO signal’s influence, we correlated each network’s signature BOLD signal with the subject’s own concurrent peripheral data (i.e., Δ[HbO] of the fingertip). The networks with high correlations are the ones being significantly influenced by the sLFOs. The results showed that in addition to clearly vascular components, such as the superior sagittal sinus, several sensory networks (i.e., visual, auditory, etc.) are strongly affected by the sLFOs (Figure 2).

### The Dynamic Nature of sLFOs in Resting State Networks

**FIGURE 3:**
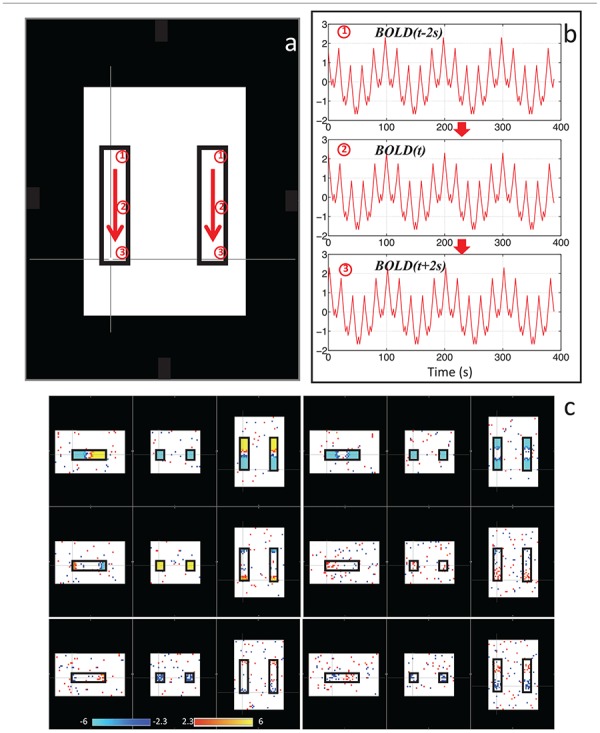
Synthetic data consisting of progressively delayed sum of sinusoids was placed inside two identical blocks **(a)**. The red arrows indicate the direction of the moving wave (increasing time delay). The examples of moving waves at the circles (1–3) are shown in panel **(b)**. Six independent components resulting from ICA are shown in panel **(c)** with the corresponding color bars (Figure adapted from Tong et al., 2015).

With this in mind, we sought to determine if the dynamic nature of the sLFO signal in BOLD could, on its own, lead to artifactual connectivity in “*Can apparent network patterns be generated by the moving physiological sLFO signal alone?*” (Tong et al., 2015). First, we performed a simple test on simulated data, which consisted of a sinusoidal wave with gradually increased time delay along the traveling direction (Figure 3a, the direction of the arrows), representing the traveling sLFO BOLD signal in the brain, additive noise, and a constant offset (see Figure 3). We then applied a standard resting state analysis methodology, namely ICA. The result showed that multiple “networks” along the traveling direction were identified, even though the only difference between the time series of these networks is the time delay (see Figure 3c). This simulation demonstrated that methods like ICA are prone to being confounded by time delayed versions of identical signals in different voxels.

**FIGURE 4:**
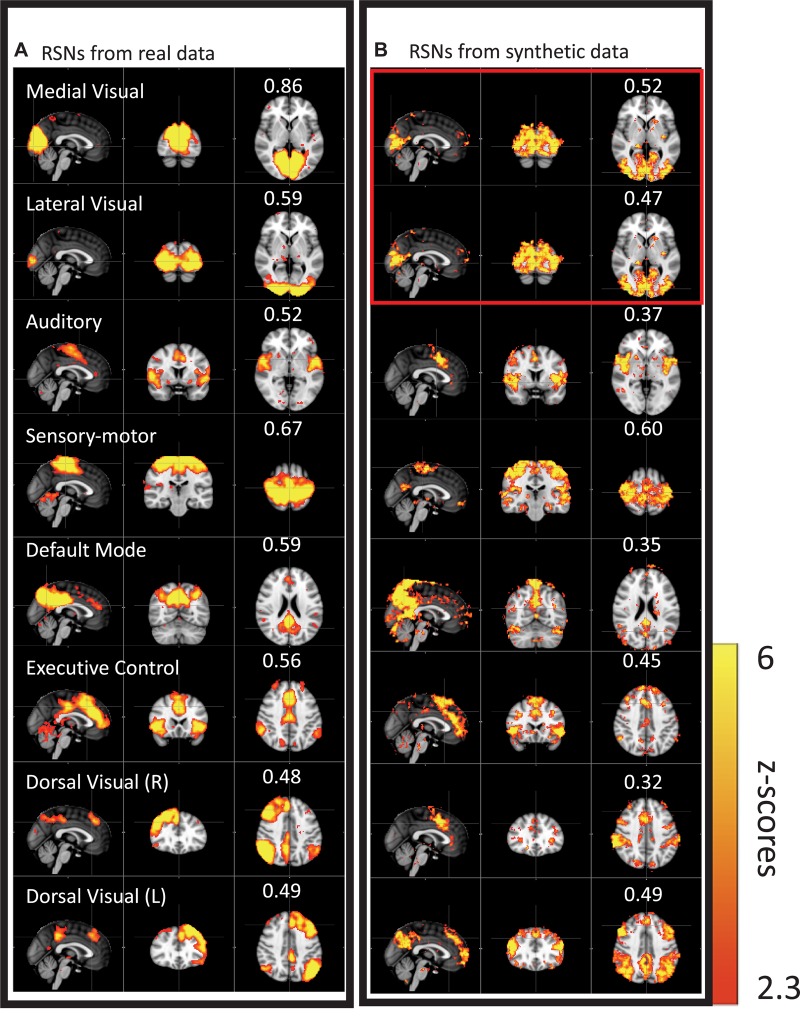
Results from group ICA on 11 subjects’ real BOLD data were shown in panel **(A)**. Results from group ICA on 11 subjects’ synthetic data were shown in panel **(B)**. The value in each result showed the spatial correlation coefficient calculated between that component and the corresponding RSN from the template (Beckmann et al., 2005). The two components in the red block are the same (Figure adapted from Tong et al., 2015).

We then performed this test on data much closer to real rsfMRI data. Initially, we calculated the delay between every brain voxel and the peripheral NIRS signal using the subjects’ real sLFO BOLD data as described above (Tong et al., 2013). Then, a sinusoidal signal, adjusted with these delays (the real delays of the subjects resting state data) replaced the real BOLD signal at each voxel (i.e., the voxel-specific time delay was decided by the delay value of that voxel). After that, we applied ICA on these simulated fMRI data with identical time series at each voxel differing only by the time delay. As a result, several “resting state networks” (RSNs) were identified (see Figure 4), some of which closely matched standard networks described in the literature. Finally, we applied seed analysis on the same simulated data and were able to identify “RSNs” as well (it is known that seed analysis is sensitive to time delays). This study demonstrated that physiological noise signals, depending only on vascular time delays can generate network patterns similar to well-known RSNs through common analytical procedures.

While clearly worrisome, these results should not be interpreted as suggesting that RSNs are nothing but a vascular artifact. There is extensive evidence for the existence of neuronal RSNs, from both animal and human studies, with a range of imaging technologies (Martinez-Montes et al., 2004; Hillman et al., 2007; Brookes et al., 2011; Ma et al., 2016). Moreover, while we have shown that up to 30% of the low frequency gray matter variance (13% of the total variance across *all* frequency bands, Hocke et al., 2016) is due to non-neuronal sLFOs (Frederick et al., 2012a), this means, necessarily, that 70% of the variance *is not* due to sLFOs, and likely represents neuronal signal. However, it is clear that there are both vascular and neuronal “connectivity” networks, with significant spatial overlap.

In order to support the metabolic demands of neurons, vascular networks are formed according to various factors, such as neuron density and metabolic demand. Areas of the brain which routinely coactivate likely develop similar vascular supplies, at similar times. This could lead to overlapping of both networks. It is also possible that, despite being a map of sLFO arrival time, the delay map may nevertheless contain some neuronal information. We hypothesize that elevated neuronal activation within a network will increase the blood flow locally, minimizing the time delays within the network. These subtle differences could be identified by ICA. Nevertheless, we demonstrated that sLFO signals will confound the quantification of some RSNs, in both the spatial and temporal domains, unless some steps are taken to disentangle these signals.

## Mitigation Strategies

As discussed, these widespread, correlated, non-neuronal sLFOs obscure and distort the more localized patterns of neuronal correlations between interacting brain regions, and may even cause apparent connectivity between regions with no neuronal interaction – numerous vascular “networks” are commonly seen in data driven connectivity analyses. Standard mitigation methods such as global signal regression (GSR) have serious drawbacks, and may in fact induce artifactual negative correlations between brain regions. Newer methods such as CompCor (Behzadi et al., 2007) avoid some of these problems, but may not fully remove the sLFO signal. By examining the structure of temporal cross-correlations with non-zero time delays throughout the brain (or by making simultaneous, independent measurements in the periphery), it is possible to parse the global, moving, blood-borne signal from the stationary, neuronal connectivity signals. In the following we describe a simple method to remove this signal, leaving the neuronal connectivity intact, while substantially reducing the negative (potentially spurious) correlations that result from global signal regression.

We have conducted continuous research on isolating, characterizing, and separating the neuronal LFO and sLFO in the resting state BOLD fMRI during the past 8 years (Tong and Frederick, 2010, 2012, 2014a, b; Tong et al., 2011a,b,c, 2012, 2013, 2014, 2015, 2016, 2017, 2018; Frederick et al., 2012a; Erdogan et al., 2016; Hocke et al., 2016). The methods developed utilized the key differences between these two oscillations, part of which are discussed in “Characteristics of LFOs”: (1) neuronal LFO is regional, while sLFO is global; (2) the neuronal LFO signal does not “propagate” in space, while sLFO does (sLFOs are dynamic), traversing the brain on the time scale of seconds; (3) while neuronal LFO should be found largely in the capillary bed (it is known to biased toward veins), sLFO BOLD is also detected near/in the large blood vessels, especially veins as well as capillaries; (4) neuronal LFO originates in the brain, while sLFO has extracerebral origins and propagates into and through the brain with the blood. These differences do allow us to effectively parse the sLFO and neuronal components of the low frequency BOLD signal, and examine them separately using a technique we call Regressor Interpolation at Progressive Time Delays (RIPTiDe) which is described in more detail below.

### Regressor Interpolation at Progressive Time Delays (RIPTiDe)

The RIPTiDe procedure has been described in detail previously (Frederick et al., 2012a). Initially, we developed a method to determine relative blood arrival times in the voxels of a resting state fMRI (rsfMRI) dataset by using simultaneous NIRS to estimate the non-neuronal, systemic signal. We use a cross-correlation procedure to determine the delay time between this peripheral signal and the timecourse of each voxel in the fMRI dataset. Determination of the precise arrival time of blood-borne signal at every brain voxel also allows correct determination of the fraction of that voxel’s signal that is accounted for by the moving blood signal, which depends on the voxel’s relative cerebral blood volume (rCBV) and the oxygenation of blood in the voxel (Tong et al., 2018). We have used this NIRS based method to remove non-neuronal signal from BOLD data (Frederick et al., 2012a), as a probe to study physiological signal partitioning in brain (Tong and Frederick, 2012, 2014a; Tong et al., 2012, 2013), and to measure cerebrovascular reserve (Tong et al., 2011a).

Our previous time-delay image analysis work focused closely on two physiological inputs – endogenous hemodynamic fluctuations in normal controls measured directly and concurrently with scanning using NIRS, and exogenous hemodynamic fluctuations (caused by a carbogen gas challenge) extracted *post hoc* from BOLD imaging data collected in symptomatic IC stenosis patients undergoing CVR experiments (Donahue et al., 2016). However, we have determined that in many cases, the signal can be extracted from the fMRI data itself, either from a region rich in venous blood (such as in the superior sagittal sinus) (Tong et al., 2016), or more simply from the global mean average of the data (Erdogan et al., 2016).

**FIGURE 5:**
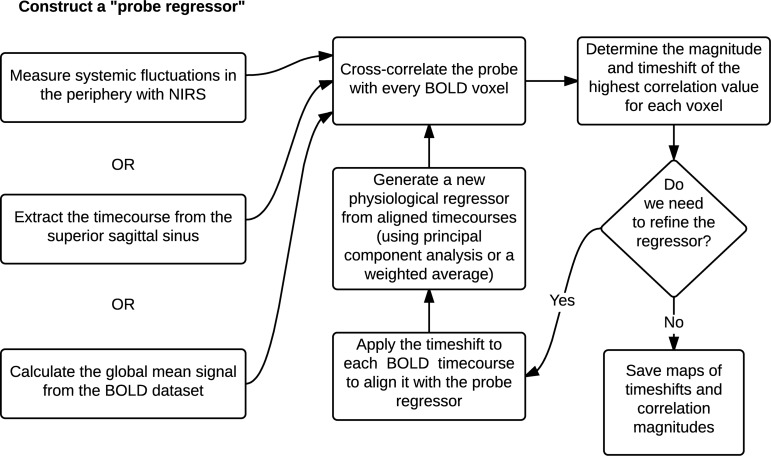
A schematic representation of the RIPTiDe regressor refinement procedure (Figure reproduced from Erdogan et al., 2016).

Using the global mean data has a number of advantages relative to other methods, the most obvious being that it requires nothing other than the fMRI data itself – no external recordings – and processing is extremely simple, as there is no need to define anatomic regions *a priori*. Moreover, we showed in our recent study (Tong et al., 2018) that the global mean signal is highly correlated with the BOLD signal extracted from the SSS (i.e., maximum cross-correlation values are 0.81 ± 0.1), which indicates that essential components of global mean overlap with those of large veins (with little neuronal contamination). The drawback is that the global mean signal is essentially a temporally “blurred” version of the physiological regressor, because it contains contributions from voxels over a range of delay values. Moreover, each voxel contains fluctuations caused by local neuronal activity (which in this case are considered noise). To overcome these drawbacks, we have developed bootstrap sharpening method to recover the source signal, which is diagrammed in Figure 5. We have released an open source software package, “rapidtide”^1^, to perform this fitting procedure and isolate and remove the sLFO signal from resting state (or task) fMRI data.

**FIGURE 6:**
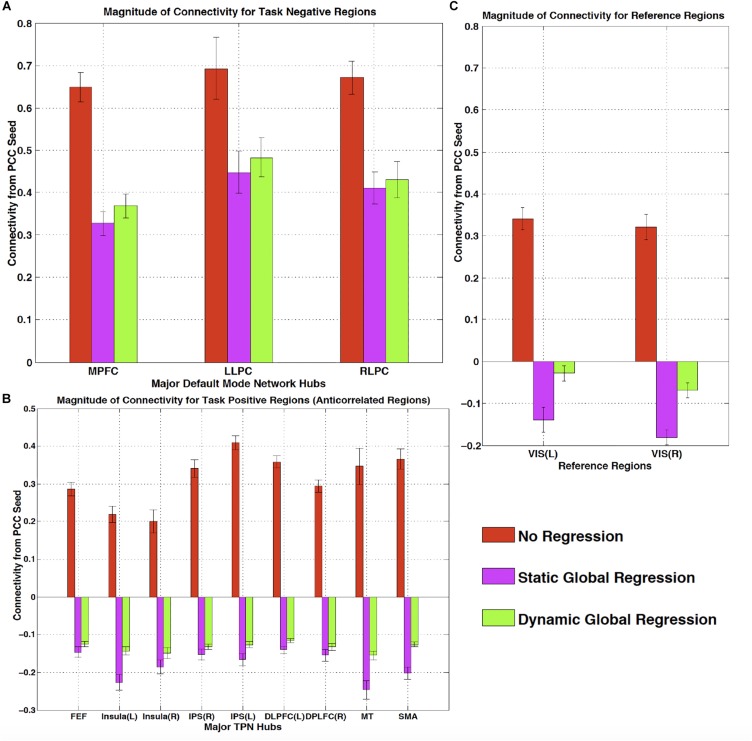
The effect of static and dynamic global signal regression on group level connectivity strengths from a posterior cingulate cortex (PCC) seed to ROIs in panel **(A)** major default mode network (DMN) ROIs (task negative regions), **(B)** Task positive network (TPN) ROIs, **(C)** and reference regions thought not to be involved in either network. L, left hemisphere; R, right hemisphere; VIS, visual cortex ROI. Both static and dynamic global signal regression remove spurious connectivity within the DMN (panel **A**), while preserving the expected anticorrelations with regions of the task positive network (panel **B**). Spurious positive correlations with unrelated reference regions were eliminated with both types of regression; however this came at the cost of large, significant spurious anticorrelations using static GSR, but not with dGSR (panel **C**) (Figure reproduced from Erdogan et al., 2016).

One of the most widespread methods of preprocessing rsfMRI to remove low frequency physiological noise is GSR. In this procedure, the mean signal of all voxels over time is regressed out of all of the BOLD time series prior to resting state analysis. While this does remove a significant amount of physiological signal, it has a serious drawback – the creation of spurious, negative correlations between brain regions (Carbonell et al., 2011). This is an unavoidable consequence of simple regression. The global mean signal is a summation of many copies of the sLFO signal with a range of delays reflecting the blood arrival time throughout the brain. Because the sLFO signal is low frequency (below 0.1–0.15 Hz), when copies of the signal over the range of delays found in the brain are summed, the signal strongly resembles the driving sLFO signal. However, this signal is not properly aligned in time in the vast majority of the voxels of the brain – it is shifted forward or backward relative to each voxels’ signal, but the correlation with each voxel is generally high. Regressing out a delayed version of the driving signal at the wrong time delay unavoidably results in a lower amplitude, inverted version of the global signal being added to the voxel at the correct time delay. This will necessarily create artifactual negative correlations in GSR processed data, as shown by Erdogan in both real and simulated data (see Figure 6, reproduced from Erdogan et al., 2016). In a detailed comparison of static global signal regresson (sGSR) with the dynamic global signal regression (dGSR) performed by rapidtide, we found that by regressing the sLFO signal out of each voxel at the proper time delays, the efficacy of noise removal was improved. More importantly, we demonstrated that by removing the sLFO dynamically, negative correlations, which were present in the results of sGSR processing, were substantially attenuated (Erdogan et al., 2016). We would argue that many of these negative correlations are potentially spurious, being generated by the removal process itself, but this has yet to be confirmed. Aso et al. (2017) have found that an analogous noise removal procedure yield similar improvements in task based analyses, and also showed significant increases in reproducibility of analyses over time.

We also compared sLFOs derived from peripheral NIRS Δ[HbO] with other LFO models (Hocke et al., 2016), namely the model-based methods for respiration and cardiac listed above in “Potential causes of the low frequency oscillation.” With high temporal resolution (TR of 400 ms), we found only small contributions (1–5%) of explained variance by the models considering respiration and cardiac variation. We also found that sLFO explained significantly more variance (up to 16%) when aliased respiration or cardiac signals do not play a critical role when fully sampled and filtered. In addition, sLFO was also substantially different from the variation models with very little temporal and spatial overlap (Hocke et al., 2016), even though NIRS Δ[HbO] is closely related to modulations in CO_2_ concentration. This study showed that sLFO is not an artificial signal created by suboptimal acquisition parameters, but a real and pervasive physiological signal accounting for a substantial amount of the variance in the BOLD LFO, and which is not accounted of by previous methods. It should be noted that this procedure cannot remove stationary non-neuronal signal. However, this type of noise, should it exist, could be removed using ICA techniques, which are well suited to detecting (and removing) spatial patterns of synchronized signals.

### Significance Determination

Because RIPTiDe analysis relies on the cross-correlation of low pass filtered signals, there is some concern that the correlations derived by the method may be spurious. Until recently, determination of the statistical significance of RIPTiDe metrics has been problematic. As the method is based on the cross-correlation of time series, it is tempting to use standard formulae, which determine significance based on number of degrees of freedom and the correlation coefficient. However, this greatly overestimates the statistical significance of the data obtained through our procedure, for two reasons. The first is that both our test regressor and the fMRI data are bandpass filtered to select the LFO component prior to correlation, which effectively reduces the degrees of freedom in the correlation. One method proposed to correct for this, specifically for the case of fMRI data, is to apply a correction factor to the degrees of freedom based on the portion of the spectrum retained by the filtering procedure (Davey et al., 2013). While this improves the estimation of significance in filtered correlations, to be strictly correct, both the exact transfer function of the filter function and the spectrum of the data being filtered must be known *a priori* and included in the calculation of the correction factor. In practice, the power spectra of fMRI data in general and the systemic low frequency oscillation signal in particular are not white (see section “Limitations” for further discussion on this topic), even over the limited frequency band of the sLFO, and vary in space. This second condition makes this procedure cumbersome.

The second, and more difficult aspect of the analysis to address, is that the peak correlation value within a range is selected to determine the “optimal” time lag, which necessarily serves to inflate the correlation value, and bias it toward more positive values. Proper application of correction for multiple comparisons requires accounting for the smoothness of the correlation function, which in turn is determined by the factors listed above. There are analytical methods for doing this (Olden and Neff, 2001), however they too are somewhat intractable for fMRI data.

While analytical calculation of the significance is difficult, there are two straightforward numerical methods to achieve this goal. The first, and simplest, method is to perform mismatched correlations. In this case the voxel timecourses are correlated with an sLFO signal from a different subject (or from the same subject at an extremely large time delay of several minutes). In this case any correlations between the timecourses are known to be spurious; a distribution of spurious correlations can be calculated to find various significance thresholds. We have employed this method in many of our analyses where the data permits (most recently here, Yao et al., 2019).

However, in cases where datasets are small, or time delayed sLFO signals are not available, there is a more general method which is also quite straightforward – we can estimate the distribution of null correlations using a Monte Carlo approach (Hocke et al., 2016). The probe regressor is permuted by randomizing the time indices, preserving the distribution of intensity values but destroying any temporal correlations, and the RIPTiDe procedure (filtering, cross-correlation, and peak finding) is performed on this timecourse with the unpermuted regressor. The procedure is repeated a sufficient number of times that the null distribution of correlation coefficients can be estimated, so the *p*-Values of different correlation coefficients can be directly determined. This procedure is rapid – our analysis software estimates this distribution from 10000 iterations at the beginning of each refinement cycle for each subject’s data in under 25 s. By default, the results presented by rapidtide are thresholded to the *p* < 0.05% level. This is probably too stringent in general, as there will be many voxels with true, lower correlation due to low rCBV. In practice for high quality data though, this is not too much of a problem.

It is important to note that for autocorrelated data, such as fMRI, one should only permute samples within exchangeability blocks to maintain the autocorrelation – time index shuffling is not generally correct. A better (but significantly slower) procedure is to randomize the phase of the Fourier transform of the data, which preserves the autocorrelation structure (Handwerker et al., 2012). However, in the case of RIPTiDe processing, both the regressor and shuffled data are filtered to the LFO band *after* shuffling, and the effect of this filter dominates the autocorrelation properties of the inputs to the correlation, so time shuffling performs well. This was verified in the HCP data, where we estimated the *p* < 0.05 correlation level both by the shuffling procedure described, and with the mismatched correlation method. The results were in close agreement, with an average spurious correlation threshold of 0.2.

## Applications of the sLfo Signal

Up to now we have discussed the moving sLFO as a noise signal contaminating rsfMRI. But whether the moving signal is noise or signal is simply a matter of perspective. If we reframe the moving, blood-borne variance as a “signal” carrying information, rather than simply a nuisance complicating the interpretation of resting state connectivity, we can use it as a sensitive measure of hemodynamic function. The relative arrival time and strength of this signal as it propagates through the cerebral vasculature carries information regarding the distribution and timing of blood flow in the brain. We propose that the moving hemodynamic signal is a unique contrast mechanism in its own right, which provides information not currently available to other techniques. It may offer insights into subtle hemodynamic alterations that can be used as early indicators of circulatory dysfunction in a number of neuropsychiatric conditions, such as prodromal stroke, moyamoya, and Alzheimer’s disease. Characterization of the sLFO signal throughout the brain allows for continuous monitoring of blood arrival time delay without a dedicated acquisition, with high sensitivity, and over a wide range of delay times, without any externally administered contrast.

### Cerebrovascular Reactivity (CVR) Mapping

The moving sLFO signal can be used to determine cerebrovascular reactivity (CVR) to CO_2_ changes both for clinical evaluation and to calibrate the BOLD response. CVR is typically measured using a hypercapnic challenge (either exogenously applied gas or breathhold) (Heyn et al., 2010; Bright and Murphy, 2013; Hare et al., 2013; Donahue et al., 2014). Accounting for the particular dynamics of the sLFO signal can give more accurate estimates of the regional response (Tong et al., 2011a). Golestani et al. (2016a, b) fully exploited the effect of the moving paCO_2_ sLFO waveform on voxel wise BOLD to perform quantitative CVR mapping throughout the brain using only resting state data. By correcting for time delay and correlating the end-tidal CO_2_ (a proxy for paCO_2_) with the BOLD signal (with additional noise removal and modeling), they were able to determine the local BOLD response per percent change in paCO_2_ simply from the resting state signal fluctuations.

### Quantitative Blood Flow Imaging

In addition to assessing CVR, sLFO signals can be used to track blood throughout the brain to reveal both normal circulation patterns, and circulatory alterations in response to tasks, pharmacological challenges, or pathology. The temporal resolution of delay measured using cross-correlation depends on the length of the input signals, rather than the signal repetition time, so very fine delay distinctions are possible with normal fMRI data. Furthermore, because the delay measurement relies on a pattern of pseudorandom signal fluctuations over the entire timecourse, rather than a single tag, such as that used in arterial spin labeling (ASL), extremely long delay times can be measured. Newer ASL techniques, such as multidelay ASL offer some information on arrival time, but are still limited by the short lifetime of the ASL tag (under 3 s). Velocity-selective ASL does remove the restrictions on tag lifetime, and has been used for delays over 6 s (Qiu et al., 2012), however, the ability to quantify delay over a range of 10 to 100 s of seconds has not been demonstrated, and this does require another scan in addition to the resting state.

#### Healthy Circulation

**FIGURE 7:**
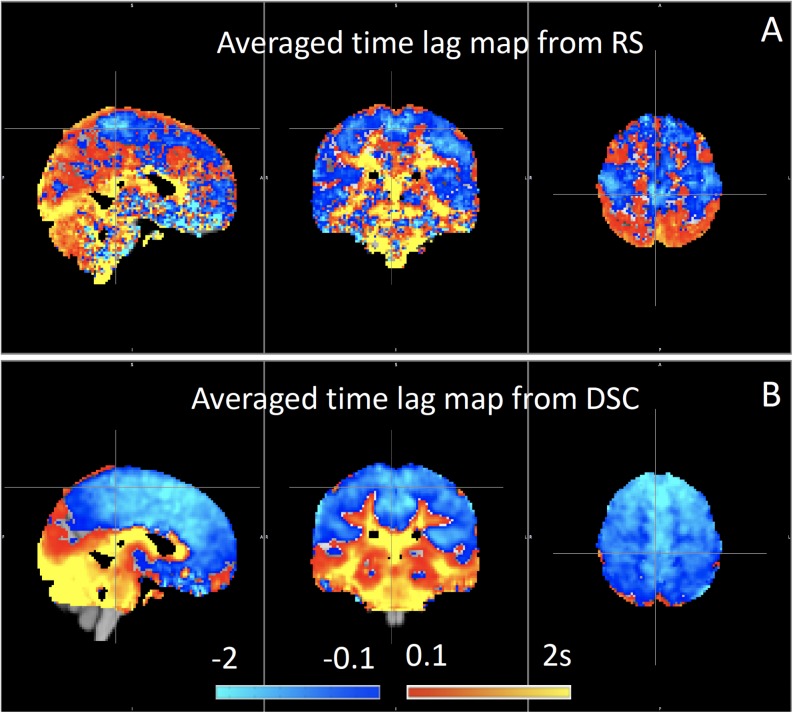
Blood arrival time delay values (in seconds) obtained from rapidtide analysis of **(A)** resting state fMRI data and from **(B)** dynamic susceptibility contrast imaging during the same imaging session in healthy controls (*N* = 8) (Figure adapted from Tong et al., 2017).

We have used RIPTiDe analysis extensively in healthy subjects to quantify typical blood flow patterns, have validated these measurements against gold standard dynamic susceptibility contrast (DSC) imaging data (see Figure 7) collected in the same session (Tong et al., 2017), as shown in Figure 7, and have followed signal through the head from the inflowing carotid arteries to the exiting jugular veins (Tong et al., 2018) to establish that the sLFO signal does indeed move with the blood.

**FIGURE 8:**
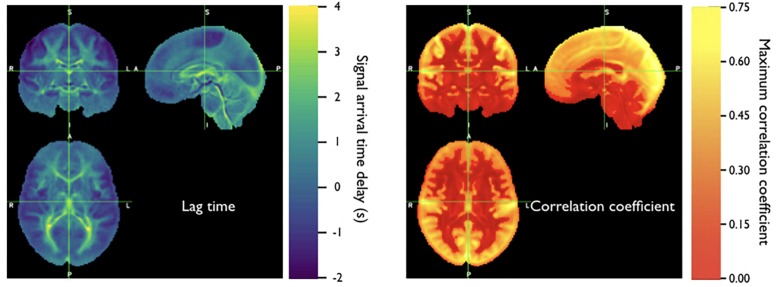
Averaged correlation parameters (lag time of maximum correlation and maximum correlation value) for 487 subjects from the 500 subjects release of the Human Connectome Project data. Each subject had four scans (LR and RL phase encode in two sessions, REST1 and REST2) (Figure adapted from Frederick et al., 2017).

Because the bootstrap RIPTiDe analysis requires only fMRI data to assess hemodynamic parameters, it can be applied retrospectively to existing data [as in the myconnectome analysis described above (Tong et al., 2017), and a wider analysis of myconnectome, ABCD, and HCP data (Yao et al., 2019)]. We have also applied this technique to the resting state data from the Human Connectome Project (Frederick et al., 2017), and were able to produce very detailed maps of average time delay and correlation strength throughout the brain (shown in Figure 8). This can provide a standard comparison dataset for young healthy controls.

It is important to note that the time delay measurement is always relative. Our standard is to set “zero time” at the peak of the histogram of delay values. As a result, there are necessarily always positive and negative delay times – positive delays tend to be in the later parts of the vascular tree – large draining veins, etc., while negative delays correspond to parts of the brain close to the source arteries (so they get blood before most other regions). This is a somewhat arbitrary choice – we could (and have) used an anatomical reference region such as the cerebellum as the delay time origin (Donahue et al., 2016) depending on the application.

We note that we consistently observe higher positive delay values in white matter (by a few seconds) than in the cortex; presumably this is because gray matter circulation is prioritized. This observation has been confirmed with DSC imaging, the gold standard MR technique for perfusion measurement.

#### Circulatory Pathology

This technique is not limited to studying healthy circulation, however. Compromised circulation due to arterial occlusion from to atherosclerosis, moyamoya disease, or stroke, can lead to extremely long delay times (up to 10 s of seconds) which are not quantifiable using conventional methods such as ASL or CT angiography. We demonstrated that time delay imaging with exogenous CO_2_ manipulation can reveal and accurately quantitate extremely long delays *in vivo* (Frederick et al., 2013; Donahue et al., 2016). However, the exogenous CO_2_ manipulation is not necessary – the hemodynamic delays arising from moyamoya disease can also be quantified using the endogenous sLFO signal from rsfMRI alone as a probe (Christen et al., 2014). Others have used the endogenous sLFO in a number of clinical applications.

#### Stroke

There have been several studies using global signal correlation delays to investigate hypoperfusion acutely and longterm after strokes. Lv et al. (2013) showed that “significant delay in BOLD signal corresponded to areas of hypoperfusion identified by contrast-based perfusion MRI” in 11 subjects acutely after ischemic stroke. Amemiya et al. (2014) found similar agreement in the results of BOLD delay and contrast perfusion measurements in five patients with chronic hypoperfusion and six with acute stroke. In 2016, Siegel assessed 130 subjects 2 weeks, and 3 and 12 months post-stroke onset (and 30 controls), and showed that increased BOLD lag delay was strongly correlated with decreased blood flow assessed with ASL. Furthermore, they found that removing the delayed hemodynamic signal somewhat normalized functional connectivity measurements (which were distorted by delayed hemodynamics) (Siegel et al., 2016). Khalil et al. (2017) assessed delay changes of sLFO BOLD signals among acute stroke patients in two separate studies. In one study, they found the delay maps were highly correlated with the time-to-peak maps derived from DSC-MRI in ischemic stroke. In another longitudinal study, they found the sizes of the extended-delay regions and the corresponding delay values changed according to the vessel conditions (Khalil et al., 2018). Recently, Nishida et al. (2018) has found the delay maps from patients with the arterial occlusive disease were correlated with the CVR maps from SPECT. These multimodal studies validated the method of BOLD delay and showed its great potential in perfusion assessment of cerebrovascular disease.

#### Other Conditions

Christen et al. (2014) were able to quantify delayed blood flow in moyamoya disease using a cross-correlation technique. In addition, Yan et al. (2018) have successfully detected perfusion deficits (from BOLD delay) in patients with Alzheimer’s disease and mild cognitive impairment.

## Limitations

There are two rather significant factors that can complicate the crosscorrelation method for tracking sLFO’s – unfortunate spectral characteristics of the sLFO signal, and initializing the proper regressor to perform the calculation. These factors are discussed below.

### Spectral Characteristics

The first problem is the random nature of the sLFO signal itself. Usually this benefits us in that it allows us to find the signal throughout the brain despite whatever local signal variations are present, and to determine its time delay – the cross-correlation of the sLFO with any other random variation (such as neuronal signals) is, in general, low, and the correlation with delayed copies of itself is high and strongly peaked at the appropriate time delay. The spurious correlation threshold can be determined through permutation or comparison of correlations between subjects, as discussed above, so we can decide when correlation is “real.”

However, all of this rests on the assumption that that sLFO signal is (1) truly random, or at least not determined by factors that will influence other noise signals found in the brain, and that (2) the signal is sufficiently “white” within the band of interest. While the first condition seems to be satisfied in general, the second frequently is not. The random nature of the signal means that by chance it sometimes has undesirable spectral properties which make it less suitable for our purposes. This leads to two problems – pseudoperiodicity and non-uniform spectra. The first is a special case of the second, but is common enough to be discussed on its own.

#### Pseudoperiodicity

The first uncontrolled quantity is pseudoperiodicity. From time to time, signal energy in the 0.09–0.15 Hz band will be strongly concentrated in one or more spectral peaks. Whether this is completely random, or due to some pathological or congenital condition that affects circulation is not known – it seems for the most part to be purely by chance, as it is occasionally seen when looking at multiple runs in the same subject, where one run is pseudoperiodic while the rest are not.

The effect of this is to cause the crosscorrelation between the probe signal and voxel timecourses to have more than one strong correlation peak. This means that in the presence of noise, or extreme spectral concentration of the sLFO, the wrong crosscorrelation peak can appear larger, leading to an incorrect delay estimation. This is particularly problematic if the pseudoperiod is shorter than the reciprocal of the search window (for example, if the search window for correlation peaks is between −5 and +5 s, and the sLFO has a strong spectral component at 0.1 Hz or higher, more than one correlation peak will occur within the search window). As the width of the search range increases, the spectral range of potentially confounding spectral peaks covers more of the sLFO frequency band.

##### Implications of pseudoperiodicity

The extent to which pseudoperiodicity is a problem depends on the application. In the case of noise removal, where the goal is to remove the global sLFO signal, and leave the local or networked neuronal signal variance, it turns out not to be much of a problem at all. If the sLFO signal in given voxel is sufficiently periodic that that the correctly delayed signal is indistinguishable from the signal one or more periods away, then it doesn’t matter which signal is removed – the resulting denoised signal is the same.

##### Mitigation of pseudoperiodicity

While we continue to work on fully resolving this issue, we have a number of ways of dealing with this. First of all, spectral analysis of the sLFO signal allows us to determine if the signal may be problematic. Rapidtide checks the autocorrelation function of the sLFO signal for large sidelobes with periods within the delay search window and issues a warning when these signals are present. Then after delay maps are calculated, they are processed with an iterative despeckling process analogous to phase unwrapping. The delay of each voxel is compared to the median delay of its neighbors. If the voxel delay differs by the period of an identified problematic sidelobe, the delay is constrained to “correct” value and refit. This procedure greatly attenuates, but does not completely solve, the problem of bad sidelobes. A more general solution to the problem of non-uniform spectra will likely improve the correction.

#### Non-uniform Spectra

As noted before, the pseudoperiodicity is a special case of non-uniformity within the sLFO spectral region. In addition to peaks in the power spectrum, there can be gaps, which are also problematic.

##### Implications of non-uniform spectra due to spectral gaps

Non-uniform spectra will tend to distort the crosscorrelation between the sLFO and any given voxel signal. In addition to sidelobes which lead to the periodic correlation functions discussed above, gaps in the spectrum, especially in the higher frequency regions, can lead to blurry correlation functions. Our method relies on identifying peaks in the crosscorrelation waveform – the sharpness of these peaks depends on the higher frequency portion of the sLFO power spectrum. If the energy of the sLFO is concentrated in the lower frequency portion of the LFO band, the crosscorrelation peaks become broad, which makes the estimation of the peak location less accurate in the presence of noise. This makes delay maps less accurate, and will tend to lower the correlation values closer to the spurious correlation threshold.

##### Mitigation of non-uniform spectra

The most straightforward solution to non-uniform spectra is to prewhiten the sLFO and voxel signals (effectively flattening the peaks and troughs of the magnitude spectrum, while preserving phase) prior to performing the correlation; there are numerous variants of this procedure, known as generalized crosscorrelation (Knapp and Carter, 1976; Liang et al., 2015). The methods require some tuning to determine thresholds for magnitude recovery to avoid inflating noise, but are included as options in rapidtide. However, when using the generalize crosscorrelation, the resulting maximum correlation magnitudes are difficult to interpret, as they no longer directly represent the amount of variance explained by the sLFO regressor.

### Inhomogeneous Time Delays

Finally, there is the problem of obtaining the sLFO regressor to begin with. In subjects with healthy circulation, the majority of the brain has delays which are relatively tightly clustered over a range of a few seconds; in this case the global mean is a good starting regressor. After multiple refinement passes, the regressor will converge on a stable candidate sLFO signal. However, pathology can lead to significant volumes of brain tissue with a large delay relative to the rest of the brain, but with a small spread of values around that delay. This can result in a global mean regressor which includes one or more “echoes” – strong, delayed copies of the true driving regressor.

#### Implications of Inhomogenous Delays and Mitigation

Having a regressor with multiple delayed copies of the driving sLFO signal will lead to ambiguous delay values, and will keep the refinement process from converging. While it may be possible to clean the signal using a technique analogous to echo cancelation, it is generally easier to avoid the situation to begin with by starting from a region of homogenous delay values. A number of the cited studies in pathology have used regressors derived from the superior sagittal sinus, which is easy to locate and clearly homogenous. However, its location at the end of the vascular tree means that blood in that region may have traversed multiple distinct paths to get there, which could result in multiple delay components. Our current thinking is to use a cerebellar ROI to derive the starting sLFO regressor, as circulation in that region is undisturbed in a wide range of pathologies (Donahue et al., 2016).

#### Stationarity

One assumption that has been made throughout these discussions is that the time delay in a region is constant over time, or “stationary.” While convenient, this is clearly a simplification. We know that these delays are not, in fact, completely constant over time. There are slight variations in delay time within HCP subjects between runs on the same day, and larger variations between days. There is no reason to suspect that there are not variations within runs as well – the correlation delays presented are averaged over the entire run. Moreover, when we calculate the delay maps from the HCP motor task data, we consistently see a regional, average 0.5 s decrease in the blood arrival time in the motor cortex relative to the values from the resting state scans, consistent with increased blood flow due to neuronal load – this is presumably an average change between the active and non-active periods leading to a shorter average delay time. This is a potential interesting area of research that as of yet, does not seem to have been explored. We have experimented some with a windowed version of RIPITiDe analysis analogous to dynamic connectivity studies, but have not worked out whether there is sufficient SNR to do this routinely.

## Conclusion

Low frequency oscillations contribute significantly to the rsfMRI signal. The signal is defined by its characteristics rather than by its origins – in specific, a low frequency range of ∼0.01–0.15 Hz. In fact, there are likely many sources of signals in this spectral region. We have discussed a number of theories for the origin of LFOs; it is important to note that these theories do not conflict – power in this frequency band is likely due to some combination of the sources we describe. Because of this, it is more useful to talk about how the low frequency contamination in the fMRI signal behaves, and what can be done about and with it.

Unlike other physiological signals, such as respiration and cardiac contamination, LFOs cannot be separated from neuronal signals through spectral filtering – they must be modeled. We have observed that up to 30% of the low frequency signal power in the gray matter moves through the vasculature, and is carried with the blood into and through the brain. This portion of the signal is therefore amenable to detection, quantitation, and removal using cross-correlation techniques, which perform well at the task of noise removal, without introducing the significant artifacts seen with other methods, such as global signal regression.

Finally, we and others have demonstrated that this moving signal can be used as a probe to quantitate cerebral hemodynamic parameters, over a wide range of conditions, without the use of contrast or specialized imaging techniques, making this an ideal method for inferring hemodynamic information both in new studies and in retrospective analysis of existing datasets. Half of these studies have been in the last 2 years, demonstrating the increasing interest in the broad clinical application of the method. As large-scale public databases such as the Human Connectome Project and the UK Biobank become available, we expect the use of these techniques to continue to expand into new research areas.

## Author Contributions

All authors listed have made a substantial, direct and intellectual contribution to the work, and approved it for publication.

## Conflict of Interest Statement

The authors declare that the research was conducted in the absence of any commercial or financial relationships that could be construed as a potential conflict of interest.

## Footnotes

1 https://github.com/bbfrederick/rapidtide

## References

[B2] Aalkjær C. Boedtkjer D. Matchkov V. (2011). Vasomotion – what is currently thought? *Acta Physiol.* 202 253–269. 10.1111/j.1748-1716.2011.02320.x 21518271

[B3] Amemiya S. Kunimatsu A. Saito N. Ohtomo K. (2014). Cerebral hemodynamic impairment: assessment with resting-state functional MR imaging. *Radiology* 270 548–555. 10.1148/radiol.13130982 24072777

[B4] Aso T. Jiang G. Urayama S. I. Fukuyama H. (2017). A Resilient, non-neuronal source of the spatiotemporal lag structure detected by BOLD signal-based blood flow tracking. *Front. Neurosci.* 11:256. 10.3389/fnins.2017.00256 28553198 PMC5425609

[B5] Beckmann C. F. Deluca M. Devlin J. T. Smith S. M. (2005). Investigations into resting-state connectivity using independent component analysis. *Philos. Trans. R. Soc. Lond. B Biol. Sci.* 360 1001–1013. 10.1098/rstb.2005.1634 16087444 PMC1854918

[B6] Behzadi Y. Restom K. Liau J. Liu T. T. (2007). A component based noise correction method (CompCor) for BOLD and perfusion based fMRI. *Neuroimage* 37 90–101. 10.1016/j.neuroimage.2007.04.042 17560126 PMC2214855

[B7] Birn R. M. Diamond J. B. Smith M. A. Bandettini P. A. (2006). Separating respiratory-variation-related fluctuations from neuronal-activity-related fluctuations in fMRI. *Neuroimage* 31 1536–1548. 10.1016/j.neuroimage.2006.02.048 16632379

[B8] Birn R. M. Smith M. A. Jones T. B. Bandettini P. A. (2008). The respiration response function: the temporal dynamics of fMRI signal fluctuations related to changes in respiration. *Neuroimage* 40 644–654. 10.1016/j.neuroimage.2007.11.059 18234517 PMC2533266

[B9] Biswal B. Yetkin F. Z. Haughton V. M. Hyde J. S. (1995). Functional connectivity in the motor cortex of resting human brain using echo-planar MRI. *Magn. Reson. Med.* 34 537–541. 10.1002/mrm.1910340409 8524021

[B10] Bright M. G. Murphy K. (2013). Reliable quantification of BOLD fMRI cerebrovascular reactivity despite poor breath-hold performance. *Neuroimage* 83 559–568. 10.1016/j.neuroimage.2013.07.007 23845426 PMC3899001

[B11] Brookes M. J. Woolrich M. Luckhoo H. Price D. Hale J. R. Stephenson M. C. (2011). Investigating the electrophysiological basis of resting state networks using magnetoencephalography. *Proc. Natl. Acad. Sci. U.S.A.* 108 16783–16788. 10.1073/pnas.1112685108 21930901 PMC3189080

[B12] Buxton R. B. Wong E. C. Frank L. R. (1998). Dynamics of blood flow and oxygenation changes during brain activation: the balloon model. *Magn. Reson. Med.* 39 855–864. 10.1002/mrm.1910390602 9621908

[B13] Carbonell F. Bellec P. Shmuel A. (2011). Global and system-specific resting-state fMRI fluctuations are uncorrelated: principal component analysis reveals anti-correlated networks. *Brain Connect.* 1 496–510. 10.1089/brain.2011.0065 22444074 PMC3604782

[B14] Chang C. Cunningham J. P. Glover G. H. (2009). Influence of heart rate on the BOLD signal: the cardiac response function. *Neuroimage* 44 857–869. 10.1016/j.neuroimage.2008.09.029 18951982 PMC2677820

[B15] Chang C. Metzger C. D. Glover G. H. Duyn J. H. Hans-Jochen H. Walter M. (2013). Association between heart rate variability and fluctuations in resting-state functional connectivity. *Neuroimage* 68 93–104. 10.1016/j.neuroimage.2012.11.038 23246859 PMC3746190

[B16] Christen T. Jahanian H. Ni W. W. Qiu D. Moseley M. E. Zaharchuk G. (2014). Noncontrast mapping of arterial delay and functional connectivity using resting-state functional MRI: a study in Moyamoya patients. *J. Magn. Reson. Imaging* 41 424–430. 10.1002/jmri.24558 24419985 PMC4096618

[B17] Crandell D. Moinuddin M. Fields M. Friedman B. Robertson J. (1973). Cerebral transit time of 99mtechnetium sodium pertechnetate before and after cerebral arteriography. *J. Neurosurg.* 38 545–547. 10.3171/jns.1973.38.5.0545 4711624

[B18] Cui X. Bray S. Bryant D. M. Glover G. H. Reiss A. L. (2011). A quantitative comparison of NIRS and fMRI across multiple cognitive tasks. *Neuroimage* 54 2808–2821. 10.1016/j.neuroimage.2010.10.069 21047559 PMC3021967

[B19] Davey C. E. Grayden D. B. Egan G. F. Johnston L. A. (2013). Filtering induces correlation in fMRI resting state data. *NeuroImage* 64 728–740. 10.1016/j.neuroimage.2012.08.022 22939874

[B20] Donahue M. J. Dethrage L. M. Faraco C. C. Jordan L. C. Clemmons P. Singer R. (2014). Routine clinical evaluation of cerebrovascular reserve capacity using carbogen in patients with intracranial stenosis. *Stroke* 45 2335–2341. 10.1161/STROKEAHA.114.005975 24938845 PMC4118584

[B21] Donahue M. J. Strother M. K. Lindsey K. P. Hocke L. M. Tong Y. Frederick B. D. (2016). Time delay processing of hypercapnic fMRI allows quantitative parameterization of cerebrovascular reactivity and blood flow delays. *J. Cereb. Blood Flow Metab.* 36 1767–1779. 10.1177/0271678x15608643 26661192 PMC5076782

[B22] Erdogan S. B. Tong Y. Hocke L. M. Lindsey K. P. de B. F. B. (2016). Correcting for blood arrival time in global mean regression enhances functional connectivity analysis of resting state fMRI-BOLD signals. *Front. Hum. Neurosci.* 10:311. 10.3389/fnhum.2016.00311 27445751 PMC4923135

[B23] Fox P. Raichle M. (1986). Focal physiological uncoupling of cerebral blood flow and oxidative metabolism during somatosensory stimulation in human subjects. *Proc. Natl. Acad. Sci. U.S.A.* 83 1140–1144. 10.1073/pnas.83.4.1140 3485282 PMC323027

[B24] Frederick B. Lindsey K. Erdogan S. Hocke L. Tong Y. (2017). “Mapping hemodynamic delay times from Human Connectome Project Resting State Data,” in *Prceedings of the 23rd Annual Meeting of the Organization for Human Brain Mapping*, Vancouver, BC.

[B25] Frederick B. Nickerson L. D. Tong Y. (2012a). Physiological denoising of BOLD fMRI data using regressor interpolation at progressive time delays (RIPTiDe) processing of concurrent fMRI and near-infrared spectroscopy (NIRS). *Neuroimage* 60 1913–1923. 10.1016/j.neuroimage.2012.01.140 22342801 PMC3593078

[B26] Frederick B. Nickerson L. Tong Y. (2012b). “Retrospective identification of global hemodynamic fluctuations from resting state fMRI,” in *Proceedings of the 3rd Biennial Resting State Brain Connectivity Conference*, Magdeburg.

[B27] Frederick B. Tong Y. (2010). “Physiological noise reduction in BOLD data using simultaneously acquired NIRS data,” in *Proceedings of the 16th Annual Meeting of the Organization for Human Brain Mapping*, Barcelona. 10.1016/j.neuroimage.2012.01.140

[B28] Frederick B. Tong Y. Strother M. Nickerson L. Lindsey K. Donahue M. (2013). “Derivation of Flow Information from a Hypocarbia Challenge Study Using Time Delay Correlation Processing,” in *Proceedings of the 21st annual meeting of the International Society for Magnetic Resonance In Medicine*, Salt Lake City, UT.

[B29] Glover G. H. Li T. Q. Ress D. (2000). Image-based method for retrospective correction of physiological motion effects in fMRI: RETROICOR. *Magn. Reson. Med.* 44 162–167. 10.1002/1522-2594(200007)44:11⁄4162::aid-mrm233⁄43.3.co;2-5 10893535

[B30] Golestani A. M. Kwinta J. B. Strother S. C. Khatamian Y. B. Chen J. J. (2016a). The association between cerebrovascular reactivity and resting-state fMRI functional connectivity in healthy adults: the influence of basal carbon dioxide. *Neuroimage* 132 301–313. 10.1016/j.neuroimage.2016.02.051 26908321 PMC5148617

[B31] Golestani A. M. Wei L. L. Chen J. J. (2016b). Quantitative mapping of cerebrovascular reactivity using resting-state BOLD fMRI: validation in healthy adults. *Neuroimage* 138 147–163. 10.1016/j.neuroimage.2016.05.025 27177763 PMC5148619

[B32] Handwerker D. A. Roopchansingh V. Gonzalez-Castillo J. Bandettini P. A. (2012). Periodic changes in fMRI connectivity. *Neuroimage* 63 1712–1719. 10.1016/j.neuroimage.2012.06.078 22796990 PMC4180175

[B33] Hare H. V. Germuska M. Kelly M. E. Bulte D. P. (2013). Comparison of CO2 in air versus carbogen for the measurement of cerebrovascular reactivity with magnetic resonance imaging. *J. Cereb. Blood Flow Metab.* 33 1799–1805. 10.1038/jcbfm.2013.131 23921896 PMC3824179

[B34] Heyn C. Poublanc J. Crawley A. Mandell D. Han J. S. Tymianski M. (2010). Quantification of cerebrovascular reactivity by blood oxygen level-dependent MR imaging and correlation with conventional angiography in patients with Moyamoya disease. *Am. J. Neuroradiol.* 31 862–867. 10.3174/ajnr.A1922 20075092 PMC7964198

[B35] Hillman E. M. C. Devor A. Bouchard M. B. Dunn A. K. Krauss G. W. Skoch J. (2007). Depth-resolved optical imaging and microscopy of vascular compartment dynamics during somatosensory stimulation. *Neuroimage* 35 89–104. 10.1016/j.neuroimage.2006.11.032 17222567 PMC1994243

[B36] Hocke L. M. Tong Y. Lindsey K. P. de B. F. B. (2016). Comparison of peripheral near-infrared spectroscopy low-frequency oscillations to other denoising methods in resting state functional MRI with ultrahigh temporal resolution. *Magn. Reson. Med.* 76 1697–1707. 10.1002/mrm.26038 26854203 PMC5796666

[B37] Hundley W. G. Renaldo G. J. Levasseur J. E. Kontos H. A. (1988). Vasomotion in cerebral microcirculation of awake rabbits. *Am. J. Physiol.* 254(1 Pt 2), H67–H71. 3337261 10.1152/ajpheart.1988.254.1.H67

[B38] Jann K. Hauf M. Kellner Weldon F. El Koussy M. Kiefer C. Federspiel A. (2016). Implication of cerebral circulation time in intracranial stenosis measured by digital subtraction angiography on cerebral blood flow estimation measured by arterial spin labeling. *Diagn. Interv. Radiol.* 22 481–488. 10.5152/dir.2016.15204 27411297 PMC5019856

[B39] Josephs O. Henson R. N. A. (1999). Event-related functional magnetic resonance imaging: modelling, inference and optimization. *Philos. Trans. R. Soc. London Ser. B Biol. Sci.* 354 1215–1228. 10.1098/rstb.1999.0475 10466147 PMC1692638

[B40] Julien C. (2006). The enigma of Mayer waves: facts and models. *Cardiovasc. Res.* 70 12–21. 10.1016/j.cardiores.2005.11.008 16360130

[B41] Khalil A. A. Ostwaldt A.-C. Nierhaus T. Ganeshan R. Audebert H. J. Villringer K. (2017). Relationship between changes in the temporal dynamics of the blood-oxygen-level-dependent signal and hypoperfusion in acute ischemic stroke. *Stroke* 48 925–931. 10.1161/STROKEAHA.116.015566 28275197

[B42] Khalil A. A. Villringer K. Filleböck V. Hu J.-Y. Rocco A. Fiebach J. B. (2018). Non-invasive monitoring of longitudinal changes in cerebral hemodynamics in acute ischemic stroke using BOLD signal delay. *J. Cereb. Blood Flow Metab.* 10.1177/0271678X18803951 [Epub ahead of print]. 30334657 PMC6928563

[B43] Knapp C. Carter G. (1976). The generalized correlation method for estimation of time delay. *IEEE Trans. Acoust. SpeechSignal Proc.* 24 320–327. 10.1109/tassp.1976.1162830

[B44] Li Y. Zhang H. Yu M. Yu W. Frederick B. D. Tong Y. (2018). Systemic low-frequency oscillations observed in the periphery of healthy human subjects. *J. Biomed. Opt.* 23 1–11. 10.1117/1.JBO.23.5.057001 29729091 PMC5935293

[B45] Liang M. Xi-Hai L. Wan-Gang Z. Dai-Zhi L. (2015). “The Generalized Cross-Correlation Method for Time Delay Estimation of Infrasound Signal,” in *Proceedings of the Fifth International Conference on Instrumentation and Measurement, Computer, Communication and Control (IMCCC)*, (Los Alamitos, CA: IEEE), 1320–1323.

[B46] Liu T. T. (2017). Reprint of ‘Noise contributions to the fMRI signal: an overview’. *Neuroimage* 154 4–14. 10.1016/j.neuroimage.2017.05.031 28647022

[B47] Logothetis N. K. Pauls J. Augath M. Trinath T. Oeltermann A. (2001). Neurophysiological investigation of the basis of the fMRI signal. *Nature* 412 150–157. 10.1038/35084005 11449264

[B48] Lv Y. Margulies D. S. Cameron Craddock R. Long X. Winter B. Gierhake D. (2013). Identifying the perfusion deficit in acute stroke with resting-state functional magnetic resonance imaging. *Ann. Neurol.* 73 136–140. 10.1002/ana.23763 23378326

[B49] Ma Y. Shaik M. A. Kozberg M. G. Kim S. H. Portes J. P. Timerman D. (2016). Resting-state hemodynamics are spatiotemporally coupled to synchronized and symmetric neural activity in excitatory neurons. *Proc. Natl. Acad. Sci. U.S.A.* 113 E8463–E8471. 10.1073/pnas.1525369113 27974609 PMC5206542

[B50] Martinez-Montes E. Valdes-Sosa P. A. Miwakeichi F. Goldman R. I. Cohen M. S. (2004). Concurrent EEG/fMRI analysis by multiway Partial Least Squares. *Neuroimage* 22 1023–1034. 10.1016/j.neuroimage.2004.03.038 15219575

[B51] Mayhew J. E. W. Askew S. Zheng Y. Porrill J. Westby G. W. M. Redgrave P. (1996). Cerebral vasomotion: a 0.1-Hz oscillation in reflected light imaging of neural activity. *Neuroimage* 4 183–193. 10.1006/nimg.1996.0069 9345508

[B52] Monti L. Donati D. Menci E. Cioni S. Bellini M. Grazzini I. (2015). Cerebral circulation time is prolonged and not correlated with EDSS in multiple sclerosis patients: a study using digital subtracted angiography. *PLoS One* 10:e0116681. 10.1371/journal.pone.0116681 25679526 PMC4334558

[B53] Müller M. W. D. Österreich M. (2014). A comparison of dynamic cerebral autoregulation across changes in cerebral blood flow velocity for 200 seconds. *Front. Physiol.* 5:327. 10.3389/fphys.2014.00327 PMC4144203 25206340

[B54] Nishida S. Aso T. Takaya S. Takahashi Y. Kikuchi T. Funaki T. (2018). Resting-state functional magnetic resonance imaging identifies cerebrovascular reactivity impairment in patients with Arterial Occlusive diseases: a pilot study. *Neurosurgery.* 10.1093/neuros/nyy434 [Epub ahead of print]. 30247676

[B55] Obrig H. Neufang M. Wenzel R. Kohl M. Steinbrink J. Einhaupl K. (2000). Spontaneous low frequency oscillations of cerebral hemodynamics and metabolism in human adults. *Neuroimage* 12 623–639. 10.1006/nimg.2000.0657 11112395

[B56] Olden J. D. Neff B. D. (2001). Cross-correlation bias in lag analysis of aquatic time series. *Mar. Biol.* 138 1063–1070. 10.1007/s002270000517

[B57] Qiu D. Straka M. Zun Z. Bammer R. Moseley M. E. Zaharchuk G. (2012). CBF measurements using multidelay pseudocontinuous and velocity-selective arterial spin labeling in patients with long arterial transit delays: comparison with xenon CT CBF. *J. Magn. Reson. Imaging* 36 110–119. 10.1002/jmri.23613 22359345 PMC3368036

[B58] Rebollo I. Devauchelle A.-D. Béranger B. Tallon-Baudry C. (2018). Stomach-brain synchrony reveals a novel, delayed-connectivity resting-state network in humans. *eLife* 7:e33321. 10.7554/eLife.33321 PMC5935486 29561263

[B59] Rivadulla C. de Labra C. Grieve K. L. Cudeiro J. (2011). Vasomotion and neurovascular coupling in the visual thalamus in vivo. *PLoS One* 6:e28746. 10.1371/journal.pone.0028746 22174886 PMC3235153

[B60] Sassaroli A. de B. F. B. Tong Y. Renshaw P. F. Fantini S. (2006). Spatially weighted BOLD signal for comparison of functional magnetic resonance imaging and near-infrared imaging of the brain. *Neuroimage* 33 505–514. 10.1016/j.neuroimage.2006.07.006 16945553

[B61] Sassaroli A. Pierro M. Bergethon P. R. Fantini S. (2012). Low-frequency spontaneous oscillations of cerebral hemodynamics investigated with near-infrared spectroscopy: a review. *IEEE J. Sel. Top. Quantum Electron.* 18 1478–1492. 10.1109/jstqe.2012.2183581 11112395

[B62] Schreiber S. J. Doepp F. Spruth E. Kopp U. A. Valdueza J. M. (2005). Ultrasonographic measurement of cerebral blood flow, cerebral circulation time and cerebral blood volume in vascular and Alzheimer’s dementia. *J. Neurol.* 252 1171–1177. 10.1007/s00415-005-0826-8 16151603

[B63] Schreiber S. J. Franke U. Doepp F. Staccioli E. Uludag K. Valdueza J. M. (2002). Dopplersonographic measurement of global cerebral circulation time using echo contrast-enhanced ultrasound in normal individuals and patients with arteriovenous malformations. *Ultrasound Med. Biol.* 28 453–458. 10.1016/s0301-5629(02)00477-5 12049958

[B64] Siegel J. S. Snyder A. Z. Ramsey L. Shulman G. L. Corbetta M. (2016). The effects of hemodynamic lag on functional connectivity and behavior after stroke. *J. Cereb. Blood Flow Metab.* 36 2162–2176. 10.1177/0271678x15614846 26661223 PMC5363662

[B65] Strangman G. Culver J. P. Thompson J. H. Boas D. A. (2002). A quantitative comparison of simultaneous BOLD fMRI and NIRS recordings during functional brain activation. *Neuroimage* 17 719–731. 10.1016/s1053-8119(02)91227-9 12377147

[B66] Tong Y. Bergethon P. R. Frederick B. D. (2011a). An improved method for mapping cerebrovascular reserve using concurrent fMRI and near-infrared spectroscopy with Regressor Interpolation at Progressive Time Delays (RIPTiDe). *Neuroimage* 56 2047–2057. 10.1016/j.neuroimage.2011.03.071 21459147 PMC3134125

[B67] Tong Y. Hocke L. M. Frederick B. (2011b). Isolating the sources of widespread physiological fluctuations in functional near-infrared spectroscopy signals. *J. Biomed. Opt.* 16:106005. 10.1117/1.3638128 22029352 PMC3210192

[B68] Tong Y. Lindsey K. P. de B. F. B. (2011c). Partitioning of physiological noise signals in the brain with concurrent near-infrared spectroscopy and fMRI. *J. Cereb. Blood Flow Metab.* 31 2352–2362. 10.1038/jcbfm.2011.100 21811288 PMC3253380

[B69] Tong Y. Frederick B. (2012). Concurrent fNIRS and fMRI processing allows independent visualization of the propagation of pressure waves and bulk blood flow in the cerebral vasculature. *Neuroimage* 61 1419–1427. 10.1016/j.neuroimage.2012.03.009 22440649 PMC3376221

[B70] Tong Y. Frederick B. D. (2010). Time lag dependent multimodal processing of concurrent fMRI and near-infrared spectroscopy (NIRS) data suggests a global circulatory origin for low-frequency oscillation signals in human brain. *Neuroimage* 53 553–564. 10.1016/j.neuroimage.2010.06.049 20600975 PMC3133965

[B71] Tong Y. Frederick B. D. (2014a). Studying the Spatial distribution of physiological effects on BOLD signals using ultrafast fMRI. *Front. Hum. Neurosci.* 8:196. 10.3389/fnhum.2014.00196 24744722 PMC3978361

[B72] Tong Y. Frederick B. (2014b). Tracking cerebral blood flow in BOLD fMRI using recursively generated regressors. *Hum. Brain Mapp.* 35 5471–5485. 10.1002/hbm.22564 24954380 PMC4206590

[B73] Tong Y. Hocke L. M. Fan X. Janes A. C. Frederick B. (2015). Can apparent resting state connectivity arise from systemic fluctuations? *Front. Hum Neurosci.* 9:285. 10.3389/fnhum.2015.00285 26029095 PMC4432665

[B74] Tong Y. Hocke L. M. Frederick B. (2014). Short repetition time multiband echo-planar imaging with simultaneous pulse recording allows dynamic imaging of the cardiac pulsation signal. *Magn. Reson. Med.* 72 1268–1276. 10.1002/mrm.25041 24272768 PMC4198428

[B75] Tong Y. Hocke L. M. Licata S. C. Frederick B. (2012). Low-frequency oscillations measured in the periphery with near-infrared spectroscopy are strongly correlated with blood oxygen level-dependent functional magnetic resonance imaging signals. *J. Biomed. Opt.* 17:106004. 10.1117/1.JBO.17.10.106004 23224003 PMC3461094

[B76] Tong Y. Hocke L. M. Lindsey K. P. Erdogan S. B. Vitaliano G. Caine C. E. (2016). Systemic low-frequency oscillations in BOLD signal vary with tissue type. *Front. Neurosci.* 10:313. 10.3389/fnins.2016.00313 27445680 PMC4928460

[B77] Tong Y. Hocke L. M. Nickerson L. D. Licata S. C. Lindsey K. P. Frederick B. (2013). Evaluating the effects of systemic low frequency oscillations measured in the periphery on the independent component analysis results of resting state networks. *Neuroimage* 76 202–215. 10.1016/j.neuroimage.2013.03.019 23523805 PMC3652630

[B78] Tong Y. Lindsey K. P. Hocke L. M. Vitaliano G. Mintzopoulos D. Frederick B. D. (2017). Perfusion information extracted from resting state functional magnetic resonance imaging. *J. Cereb. Blood Flow Metab.* 37 564–576. 10.1177/0271678x16631755 26873885 PMC5381451

[B79] Tong Y. Yao J. F. Chen J. J. Frederick B. B. (2018). The resting-state fMRI arterial signal predicts differential blood transit time through the brain. *J. Cereb. Blood Flow Metab.* 39 1148–1160. 10.1177/0271678X17753329 29333912 PMC6547182

[B80] Tsuji M. Saul J. P. du Plessis A. Eichenwald E. Sobh J. Crocker R. (2000). Cerebral intravascular oxygenation correlates with mean arterial pressure in critically Ill premature infants. *Pediatrics* 106 625–632. 10.1542/peds.106.4.625 11015501

[B81] van Beek A. H. Claassen J. A. Rikkert M. G. O. Jansen R. W. (2008). Cerebral autoregulation: an overview of current concepts and methodology with special focus on the elderly. *J. Cereb. Blood Flow Metab.* 28 1071–1085. 10.1038/jcbfm.2008.13 18349877

[B82] Wise R. G. Ide K. Poulin M. J. Tracey I. (2004). Resting fluctuations in arterial carbon dioxide induce significant low frequency variations in BOLD signal. *Neuroimage* 21 1652–1664. 10.1016/j.neuroimage.2003.11.025 15050588

[B83] Yacin S. M. Chakravarthy V. S. Manivannan M. (2011). Reconstruction of gastric slow wave from finger photoplethysmographic signal using radial basis function neural network. *Med. Biol. Eng. Comput.* 49 1241–1247. 10.1007/s11517-011-0796-1 21748397

[B84] Yan S. Qi Z. An Y. Zhang M. Qian T. Lu J. (2018). Detecting perfusion deficit in Alzheimer’s disease and mild cognitive impairment patients by resting-state fMRI. *J. Magn. Reson. Imaging* 388 (Suppl. 1), 505–506. 10.1002/jmri.26283 30318645

[B85] Yao J. F. Wang J. H. Yang H. C. S. Liang Z. Cohen-Gadol A. A. Rayz V. L. (2019). Cerebral circulation time derived from fMRI signals in large blood vessels. *J. Magn. Reson. Imaging.* 10.1002/jmri.26765 31034667 PMC7171696

[B86] Zhang R. Zuckerman J. H. Giller C. A. Levine B. D. (1998). Transfer function analysis of dynamic cerebral autoregulation in humans. *Am. J. Physiol.* 274 H233–H241. 9458872 10.1152/ajpheart.1998.274.1.h233

